# Supplementary material for: Product lambda-doublet ratios as an imprint of chemical reaction mechanism
Source: Nat Commun. 2016 Nov 11;7:13439. doi: 10.1038/ncomms13439 (PMC5114621; doi:10.1038/ncomms13439)
Supplement: Supplementary Information — Supplementary Figures 1-5, Supplementary Notes 1-2 and Supplementary References [file ncomms13439-s1.pdf]

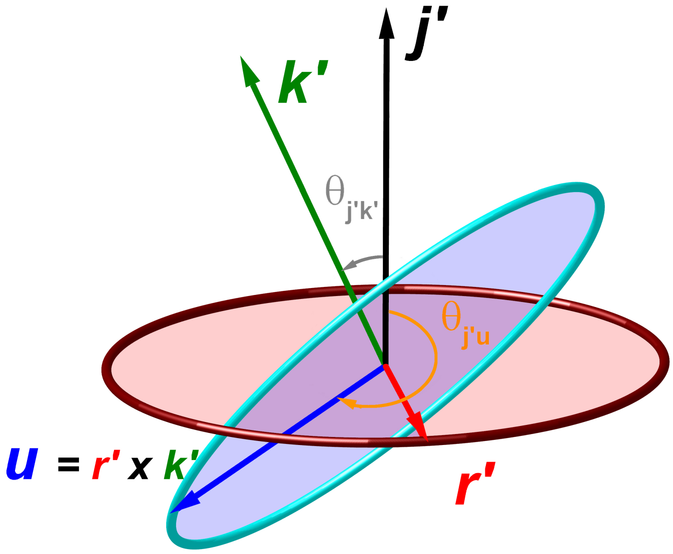

Supplementary Figure 1 . The frame of coordinates that defines the various vectors relevant in the quasiclassical description. The rotational angular momentum, $\boldsymbol{j}'$, is shown in black and kept fixed along the z axis. The O-H internuclear axis ($\boldsymbol{r}'$, in red) rotates perpendicular to $\boldsymbol{j}'$ and its possible values are shaded in red. The recoil direction $\boldsymbol{k}'$, also fixed, is shown as a green arrow. Vectors $\boldsymbol{k}'$ and $\boldsymbol{r}'$ define the three-atom plane. The possible directions of the vector $\boldsymbol{u} = \boldsymbol{r}' \times \boldsymbol{k}'$ are shaded in blue. For a particular $\boldsymbol{r}'$ (red vector), $\boldsymbol{u}$ is shown as a blue arrow.

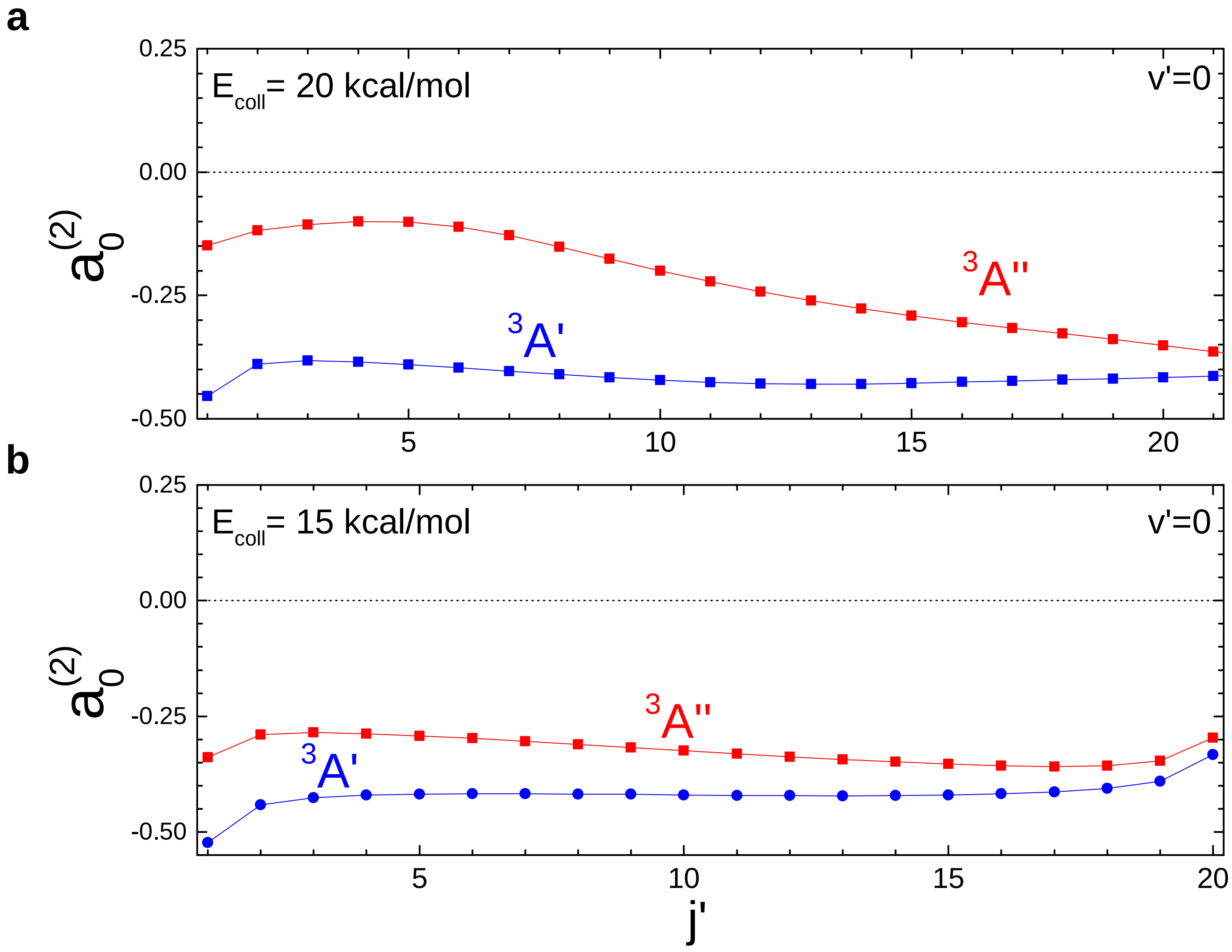

Supplementary Figure 2 . QM $a_0^{(2)}$ alignment parameter. QM product alignment parameters, $a_0^{(2)}$, referenced to $\boldsymbol{k}'$, which defines the z axis, for **(a)** $E_{\rm coll} = 20\,{\rm kcal\,mol}^{-1}$ and **(b)** $15\,{\rm kcal\,mol}^{-1}$ and the $v' = 0$ manifold. Notice that for j'=1 the QM limiting value may be lower than the classical value of $-1/2$.

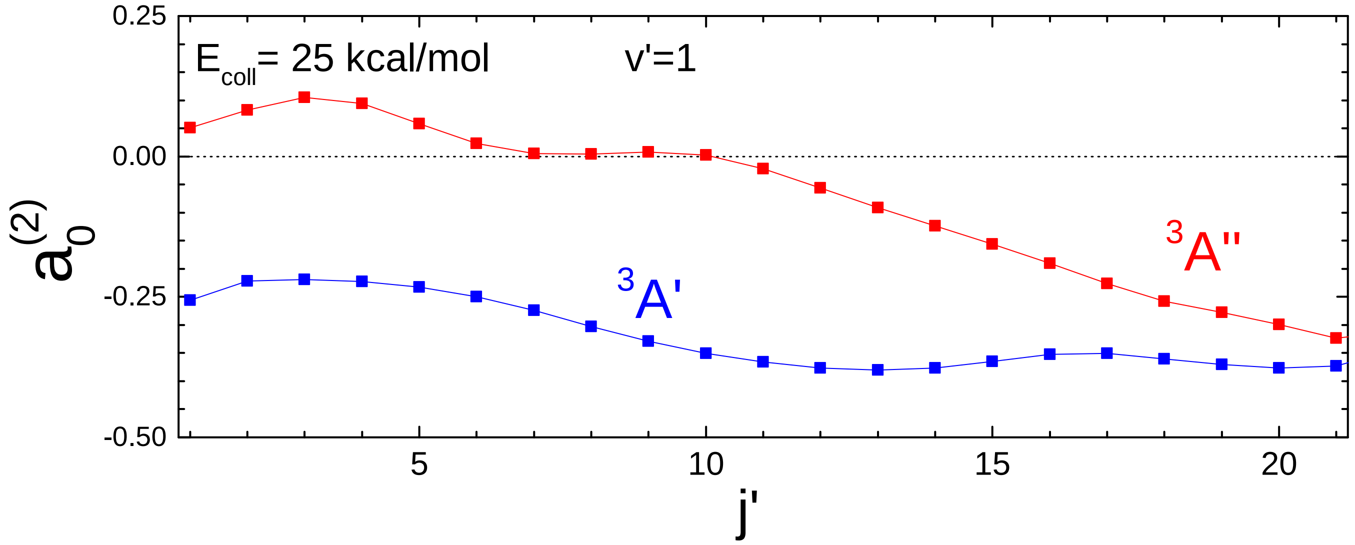

Supplementary Figure 3 . QM $a_0^{(2)}$ alignment parameter. QM product alignment parameters $a_0^{(2)}$ referenced to $\boldsymbol{k}'$, which defines the z axis, for $E_{\rm coll} = 25\,{\rm kcal\,mol^{-1}}$ and the $v' = 1$ manifold.

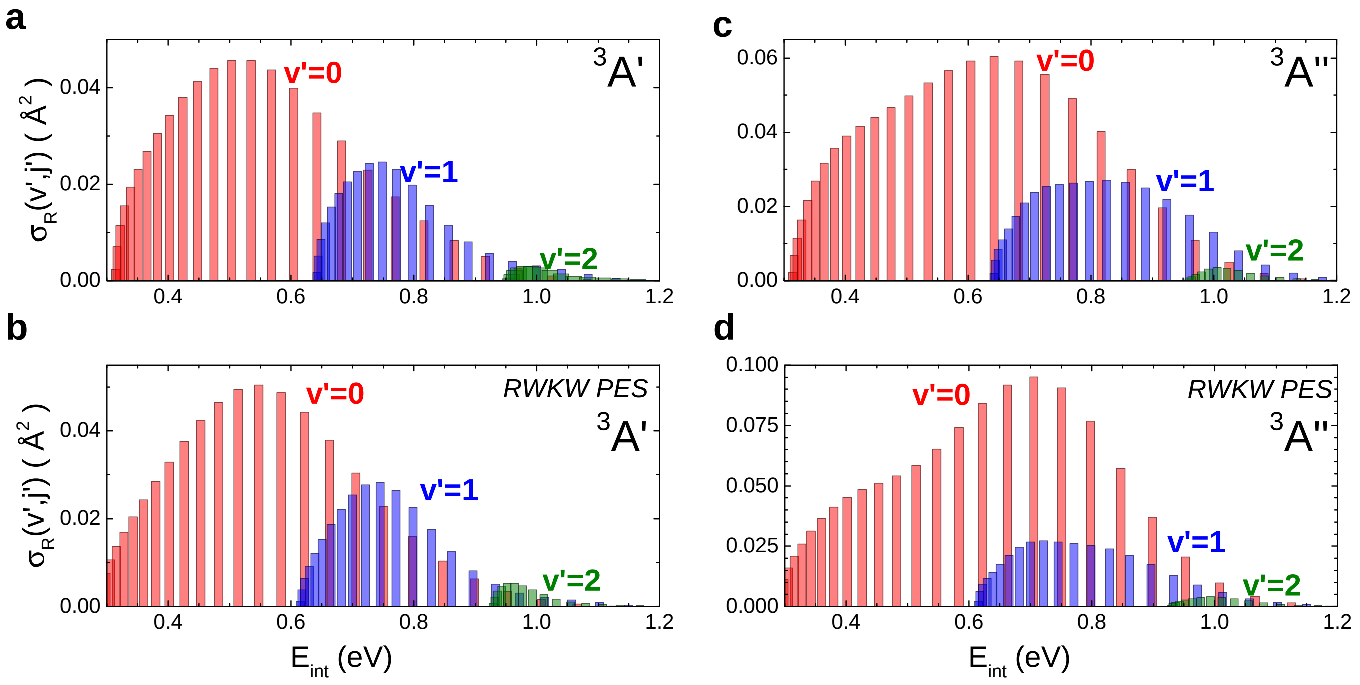

Supplementary Figure 4 . Comparison of the internal state distribution calculated on the present work and the RWKW PESs. QM state-to-state reactive cross sections calculated on the A' and A'' PESs, at $E_{\rm coll}$=25 kcal/mol as a function of the products internal energy. **(a)** results calculated on the present A' PES. **(b)** Results calculated on the RWKW A' PES. **(c)** Results calculated on the present A'' PES. **(d)** results calculated on the RWKW A'' PES. The results calculated on the RWKW PESs[1] are analogous to those presented in Ref. 2.

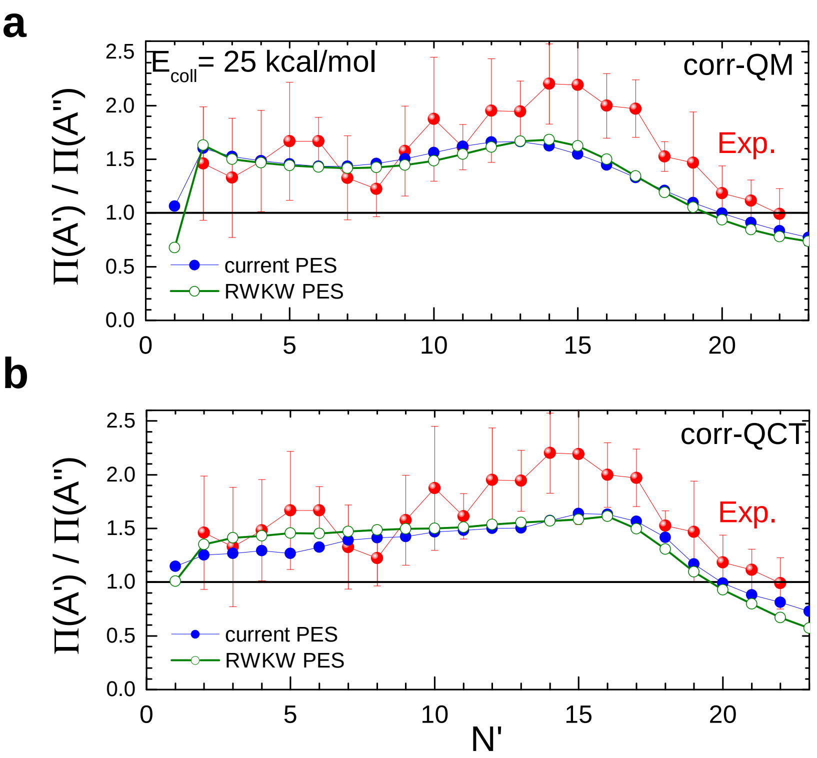

Supplementary Figure 5 . Comparison between the Λ-doublet ratios calculated on the present PESs and the RWKW PESs. **(a)** QM calculations at E_{coll}=25 kcal/mol. **(b)** QCT calculations at E_{coll}=25 kcal/mol. The experimental error bars represent the +1σ statistical uncertainties based on variations in repeated measurements.[3]

Supplementary Note 1: Polarization Parameters

As highlighted in the main article, stereodynamics (the $\boldsymbol{k}' - \boldsymbol{j}'$ correlation) plays a key role in the Λ-doublet propensity. Actually, the weight factors $W_{A'}$ and $W_{A''}$ depend only on the $a_0^{(2)}$ moments, which provide information about the alignment of $\boldsymbol{j}'$ with respect to the product recoil velocity. The value of the $a_0^{(2)}$ is given by[4]:

$$a_0^{(2)}(j') = \frac{\langle 3\hat{j}'^2_z - \hat{j}'^2 \rangle}{2\sqrt{j'(j'+1)(j'+3/2)(j'-1/2)}}\,, \tag{1}$$

where $\langle \hat{A} \rangle$ indicates the expectation value of an operator $\hat{A}$. It should be stressed that positive (negative) values of $a_0^{(2)}$ indicates an alignment of $\boldsymbol{j}'$ along (perpendicular to) $\boldsymbol{k}'$. The expectation value of the classical analogue of the $a_0^{(2)}$ is:

$$a_0^{(2)}(j') = \frac{3\langle \cos^2\theta_{\boldsymbol{j}'\boldsymbol{k}'} \rangle - 1}{2} = \langle P_2(\cos\theta_{\boldsymbol{j}'\boldsymbol{k}'}) \rangle\,, \tag{2}$$

where $P_n(x)$ is the nth-degree Legendre polynomial. This polarization parameter is equivalent to $\beta_0^0(2,2)$ defined by Dixon.[5] It is very common in the literature to defined this alignment parameter as $A_0^{(2)} = 2a_0^{(2)}$ whose classical limits will thus be -1 and +2.

As supplementary information, the values of the QM alignment moments, $a_0^{(2)}$, for OD ($v' = 0$) at $E_{\rm coll} = 20\,\mathrm{kcal\,mol^{-1}}$ and $15\,\mathrm{kcal\,mol^{-1}}$ are given in Fig. S2. The $a_0^{(2)}$ calculated at $E_{\rm coll} = 20\,\mathrm{kcal\,mol^{-1}}$ are similar to those calculated at $25\,\mathrm{kcal\,mol^{-1}}$, shown in the main text. At $E_{\rm coll} = 15\,\mathrm{kcal\,mol^{-1}}$, the values of the $a_0^{(2)}$ obtained on the A'' PES are lower (but still above the corresponding values on the A' PES). These results are not surprising since, for energies close to the classical barrier, the system tends to follow the collinear minimum energy path and similar values of $a_0^{(2)}(j')$ can be expected on both PESs.

As shown in Fig. S3, for $E_{\rm coll} = 25\,\mathrm{kcal\,mol^{-1}}$ and $v' = 1$, the $a_0^{(2)}$ values are somewhat more positive than those obtained for the $v' = 0$ manifold. It is worth mentioning that, regardless of the large difference between the values of $a_0^{(2)}$ calculated at $15\,\mathrm{kcal\,mol^{-1}}$ and those shown for $v' = 1$ and $25\,\mathrm{kcal\,mol^{-1}}$, in both cases the present theoretical predictions account fairly well for the experimental results, thus lending credence to the method used to determine the Λ-doublet state populations developed in this work.

Supplementary Note 2: Analysis of the mechanism

In this section we analyze the Supplementary Movies 1, and 2. Starting from the same set of initial collision conditions, the animations show the different outcomes of the trajectories depending on which of the two concurrent PESs is employed. Because at large $O\cdots D_2$ distances the two PESs are nearly degenerate, both trajectories are identical in the reagent valley, showing an approaching O–D–D angle of $112°$.

On the A' PES (Supplementary Movie 1) the repulsive barrier found for this O–D–D approaching angle makes it impossible for the trajectory to proceed towards products, forcing the rotation of the D_2 molecule in the scattering plane towards more collinear geometries. Only when the system reaches collinearity is the deuterium atom transferred, leading to products. The plane of rotation does not change once the reaction has been completed, so the products rotate in the scattering plane (low $|\Omega'|$), *i.e.* OD is mainly produced in a $\Pi(A')$ state.

The "cone of acceptance" on the A'' PES is significantly broader than that on the A' PES. Therefore, the deuterium transfer can take place at bent configurations at which O and D_2 initially approach one another (Supplementary Movie 2). Transfer at this bent configuration induces the OD rotation in a plane perpendicular to the scattering plane, thus leading to a $\Pi(A')$ state (recall that on the A'' PES the direction of the singly occupied orbital is perpendicular to the three atom plane).

Supplementary References

[1] Rogers, S., Wang, D., Kuppermann, A. & Walch, S. Chemically accurate ab initio potential energy surfaces for the lowest $^3A'$ and $^3A''$ electronically adiabatic states of O(^3P) + H_2. *J. Phys. Chem. A* **104**, 2308–2325 (2000).

[2] Garton, D. J. *et al.* Experimental and theoretical investigations of the inelastic and reactive scattering dynamics of O(^3P) + D_2. *J. Phys. Chem. A* **110**, 1327–1341 (2006).

[3] Lahankar, S. A., Zhang, J., McKendrick, K. G. & Minton, T. K. Product-state-resolved dynamics of the elementary reaction of atomic oxygen with molecular hydrogen, O(^3P) + D_2 $\rightarrow$ OD ($X^2\Pi$) + D. *Nat. Chem.* **5**, 315 (2013).

[4] de Miranda, M. P., Aoiz, F. J., Bañares, L. & Saéz-Rábanos, V. A unified quantal and classical description of the stereodynamics of elementary chemical reactions: state-resolved k-k'-j' vector correlation for the H+D_2(v=0, j=0) reaction. *J. Chem. Phys* **12**, 5368 (1999).

[5] Dixon, R. N. The determination of the vector correlation between photofragment rotational and translational motions from the analysis of doppler-broadened spectral line profiles. *J. Chem. Phys.* **85**, 1866 (1986).
